# Next-Generation Sequencing of RNA and DNA Isolated from Paired Fresh-Frozen and Formalin-Fixed Paraffin-Embedded Samples of Human Cancer and Normal Tissue

**DOI:** 10.1371/journal.pone.0098187

**Published:** 2014-05-30

**Authors:** Jakob Hedegaard, Kasper Thorsen, Mette Katrine Lund, Anne-Mette K. Hein, Stephen Jacques Hamilton-Dutoit, Søren Vang, Iver Nordentoft, Karin Birkenkamp-Demtröder, Mogens Kruhøffer, Henrik Hager, Bjarne Knudsen, Claus Lindbjerg Andersen, Karina Dalsgaard Sørensen, Jakob Skou Pedersen, Torben Falck Ørntoft, Lars Dyrskjøt

**Affiliations:** 1 Department of Molecular Medicine (MOMA), Molecular Diagnostic Laboratory, Aarhus University Hospital, Skejby, Aarhus, Denmark; 2 AROS Applied Biotechnology A/S, Science Park Skejby, Aarhus, Denmark; 3 CLC bio, Aarhus, Denmark; 4 Institute of Pathology, Aarhus University Hospital, Aarhus, Denmark; UT MD Anderson Cancer Center, United States of America

## Abstract

Formalin-fixed, paraffin-embedded (FFPE) tissues are an invaluable resource for clinical research. However, nucleic acids extracted from FFPE tissues are fragmented and chemically modified making them challenging to use in molecular studies. We analysed 23 fresh-frozen (FF), 35 FFPE and 38 paired FF/FFPE specimens, representing six different human tissue types (bladder, prostate and colon carcinoma; liver and colon normal tissue; reactive tonsil) in order to examine the potential use of FFPE samples in next-generation sequencing (NGS) based retrospective and prospective clinical studies. Two methods for DNA and three methods for RNA extraction from FFPE tissues were compared and were found to affect nucleic acid quantity and quality. DNA and RNA from selected FFPE and paired FF/FFPE specimens were used for exome and transcriptome analysis. Preparations of DNA Exome-Seq libraries was more challenging (29.5% success) than that of RNA-Seq libraries, presumably because of modifications to FFPE tissue-derived DNA. Libraries could still be prepared from RNA isolated from two-decade old FFPE tissues. Data were analysed using the CLC Bio Genomics Workbench and revealed systematic differences between FF and FFPE tissue-derived nucleic acid libraries. In spite of this, pairwise analysis of DNA Exome-Seq data showed concordance for 70–80% of variants in FF and FFPE samples stored for fewer than three years. RNA-Seq data showed high correlation of expression profiles in FF/FFPE pairs (Pearson Correlations of 0.90 +/- 0.05), irrespective of storage time (up to 244 months) and tissue type. A common set of 1,494 genes was identified with expression profiles that were significantly different between paired FF and FFPE samples irrespective of tissue type. Our results are promising and suggest that NGS can be used to study FFPE specimens in both prospective and retrospective archive-based studies in which FF specimens are not available.

## Introduction

Formalin-fixed, paraffin-embedded (FFPE) tissue samples stored in diagnostic pathology archives represent an invaluable biobank for retrospective clinical research. In addition, FFPE specimens may be useful in prospective studies omitting the collection of fresh-frozen (FF) specimens. Unfortunately, nucleic acids are more difficult to extract from FFPE tissue because of the need to remove the paraffin and to counteract covalent protein-DNA interactions that result from the fixation process. In addition, fixation delay (i.e. perioperative ischemic time), the fixation process, tissue preparation, paraffin embedding, and archival storage contribute to fragmentation, cross-linking and chemical modification of FFPE tissue-derived nucleic acids. These changes interfere with many classical molecular analyses requiring high quality nucleic acids. Advances in next-generation sequencing (NGS) technologies allow the investigation of genomes, epigenomes and transcriptomes using limited sample material. Moreover, this analysis can be made at a relatively modest cost, considering the massive increase in the amount of information that may be obtained. The power of NGS to analyse in depth large numbers of short sequences potentially makes this an ideal technology to apply to the usually fragmented nucleic acids that may be extracted from FFPE specimens. The development of reliable NGS-based methods for use with low-quality FFPE tissue-derived nucleic acids would open the diagnostic pathology archives to high-throughput profiling, facilitating extensive retrospective clinical studies. Similarly, the ability to use FFPE samples for molecular analysis in prospective studies would be of great benefit, by potentially reducing or even eliminating the need for the tedious collection and storage of cryopreserved clinical samples.

While nucleic acids isolated from FFPE tissues have previously mainly been studied using PCR- and microarray-based methods, there are now reports on the application of NGS to FFPE material. One of the first came from Schweiger *et al.* who reported NGS-based analysis of DNA (DNA-Seq) isolated from FFPE samples [1]. The authors found a good correlation between matched FF and FFPE breast cancer samples from a single patient when analysing genome-wide copy number alterations and variant frequencies based on low-coverage whole-genome sequencing [1]. Subsequently, Wood *et al.* combined pooling of samples and reduction in the amount of input DNA to study genome-wide copy number alterations in FFPE samples [2]. A further paper from Schweiger's group showed that studies of genomic variations based on sequencing of DNA from FFPE tissue benefited from the increased coverage obtained using targeted resequencing, reducing the impact of the fixation-induced noise [3]. Recently, Tuononen *et al.* applied targeted DNA resequencing to screen FFPE non-small cell lung carcinomas for clinically important *EGFR*, *KRAS* and *BRAF* mutations and found a significant correlation with the results of real-time PCR based analyses performed in parallel [4]. Spencer *et al.* applied targeted resequencing of 27 cancer related genes to 16 FF/FFPE paired lung adenocarcinomas and found, that in spite of demonstrable effects of the fixation process, identical single-nucleotide variants could be reliably detected [5]. Fanelli *et al.* have reported a high-throughput sequencing method for epigenetic profiling based on ChIP-seq, FFPE samples from a mouse leukemia model and from a limited range of human cancers (one seminoma and six breast carcinomas), that allow analysis of histone modifications and transcription factor binding on a genome-wide scale [6], [7]. Gu *et al.* [8], [9] showed that low input amounts FFPE tissue-derived DNA could be used together with reduced representation bisulfite sequencing (RRBS) to allow studies of genome-wide DNA methylation profiles on archival clinical samples.

Weng *et al.* were one of the first to describe NGS-based analysis of RNA (RNA-Seq) isolated from FFPE samples [10]. These authors reported comparable expression results from deep sequencing of miRNAs derived from paired FF and FFPE renal cell carcinomas. Furthermore, they found a high correlation comparing the results of NGS with those obtained in parallel using microarray and RT–PCR methodologies. Recently, Meng *et al.* reported the successful application of ligation-based miRNA sequencing to cancer samples stored up to 9 years [11]. While it is known from studies using traditional molecular techniques that smaller RNA molecules such as miRNA are better able to withstand formalin fixation [12], there is a general expectation that longer RNA molecules are likely to be more susceptible to fragmentation and modifications, thus making them poor templates for RNA-Seq. A NGS-based study of longer RNA molecules has been reported by Sinicropi *et al.* who found that RNA-Seq data derived from FFPE primary breast cancers was of sufficient quality to enable biomarker discovery [13]. Adiconis *et al.* reported a comparative analysis of five methods for RNA-Seq applied to low-quality RNA (such as that isolated from FFPE tissue) and low-quantity RNA [14]. Recently, Norton *et al.* applied RNA-Seq to nine sets of FF and FFPE breast tumour samples stored for up to four years and found good correlation between the paired FF and FFPE expression profiles [15].

We expand on these studies in terms of number and diversity of human specimens investigated and reports a systematic analysis of the potential use of NGS-based profiling of human FFPE samples in retrospective and prospective clinical studies. Our study includes (1) the evaluation of different isolation strategies of nucleic acids from human FFPE specimens and matching FF and FFPE specimens; (2) the preparation of targeted genome (DNA Exome-Seq) and whole transcriptome (RNA-Seq) sequencing libraries; and (3) thorough analysis of the NGS data obtained. The primary objectives were to identify and characterize systematic effects of the fixation process on the results obtained as well as critical parameters in the workflow from surgery to analysed NGS data. Available extraction kits were evaluated with respect to purification of RNA and DNA from selected FFPE human specimens (normal liver, liver carcinoma, reactive tonsil, lung carcinoma, breast carcinoma, normal skin from breast, and bladder carcinoma; with matching FF samples from the liver and tonsil samples). This was followed by evaluation of the extraction of RNA and DNA from FFPE human specimens (colorectal carcinoma, normal liver, bladder carcinoma, and tonsil) with different routine archival storage times (up to 244 months). Finally, DNA and RNA were extracted from matching FF and FFPE human specimens (bladder, prostate and colon carcinomas as well as from normal colon tissue). Extracted DNA and RNA were used for the preparation of targeted genome (DNA Exome-Seq) and whole transcriptome (RNA-Seq) sequencing libraries and NGS data obtained was analysed, focusing on the discovery of systematic effects of the fixation process.

## Methods and Materials

### Ethics statements

All experiments in this study were conducted on specimens of human origin selected from the frozen tissue biobanks at Aarhus University Hospital, Denmark and from the diagnostic FFPE block archive of the Institute of Pathology, Aarhus University Hospital, Denmark. The tissues were removed in the course of routine surgical procedures and were surplus to diagnostic requirements. Samples were anonymized before use in this study. The study was approved by the Committee on Health Research Ethics of the Central Denmark Region and the Danish Data Protection Agency.

### Clinical samples and major objectives

Study workflows from surgery to final analysis of the NGS data are illustrated in Figure S1 together with the key parameters investigated. The samples were selected to maximize the range of variables (e.g. storage time since collection and tissue type) with an expected impact on the results. The sample sets are described in this section, in Table 1 and in Table S1. The study included in total 23 FF, 35 FFPE and 38 paired FF/FFPE specimens, representing six different human tissue types (bladder, prostate and colon carcinoma; liver and colon normal tissue; reactive tonsil). A total of 16 FF and FFPE specimens from different tissues were selected for initial testing of DNA and RNA purification (*the test set*). To study the impact of tissue type and storage time after fixation, FFPE blocks from four tissues (colorectal and bladder carcinoma, and normal liver and tonsil) were selected, each with four storage times (2–3, 13–15, 60–62 and 241–244 months). This experiment is denoted *the storage time FFPE set*. The largest experiment, *the paired FF/FFPE colon set*, included 19 paired FF/FFPE colorectal tumour samples (both benign and malignant) and 13 FF samples of matching normal colon tissue that had been collected 2 – 13 years previously. To test the detection of specific single nucleotide variants, a set of 11 colon samples were selected with known variants in *KRAS* and *BRAF* (*the colon KRAS/BRAF variants detection set*). Furthermore, seven paired FF/FFPE prostate carcinoma samples collected 2 – 11 years previously (*the paired FF/FFPE prostate set*), eight paired FF/FFPE bladder carcinoma samples collected 5 – 9 years previously (*the paired FF/FFPE bladder set*) and 10 FF bladder carcinoma samples (*the FF bladder signature conservation set*) were selected for study. DNA and RNA were isolated from each sample and used to generate libraries for exome sequencing and total-RNA sequencing. The primary objective of the DNA Exome-seq experiments was to compare the detection of genomic variation in matched FF and FFPE samples, including both specific variants of clinical relevance as well as global variations. The RNA-Seq experiments were conducted to allow comparisons to be made of the transcriptional information obtained from matched FF and FFPE samples, including (1) global information on transcriptional activity; (2) specific expression levels in relation to biological status (i.e. contrasting benign and malignant samples in *the paired FF/FFPE colon set*); and (3) description of an expression signature for bladder tumour progression (in *the FF bladder signature conservation set* and *the paired FF/FFPE bladder set*).

**Table 1:** Overview of the different experiments included in this study.

Experiment	Samples
*The test set*	A total of 16 samples from normal liver, normal (reactive) tonsil, normal skin (from mastectomy specimen), liver, lung, breast, and bladder carcinomas. Matching FF samples were available from the liver and tonsil samples.
*The storage time FFPE set*	Four FFPE tissues (normal liver and tonsil; colorectal and bladder carcinomas), each from four storage time points (2–3, 13–15, 60–62 and 241–244 months).
*The paired FF/FFPE colon set*	19 paired FF/FFPE colorectal carcinoma samples; in 13 of these, matching normal FF colon samples. This set had been stored for 2 – 13 years.
*The colon KRAS/BRAF variants detection set*	11 FFPE colon carcinoma samples.
*The paired FF/FFPE prostate set*	7 paired FF/FFPE prostate carcinoma samples, stored for 2 – 11 years.
*The paired FF/FFPE bladder set*	8 paired FF/FFPE bladder carcinoma samples, stored for 5 – 9 years.
*The FF bladder signature conservation set*	10 FF samples of Ta bladder carcinoma, 3 showed later progression.

### Purification of DNA and RNA from FF and FFPE tissue

To identify extraction methods resulting in optimal nucleic acid quality and yield, RNA and DNA were purified from the FFPE blocks of the *test set* (Table 1 and Table S1) using commercially available kits as described below. Up to six 10 µm sections were cut from each tumour block and placed in sterile 1.5 mL centrifuge tubes ready for extraction. Tubes containing cut FFPE sections for RNA purification were stored at −80°C until use. DNA and RNA purifications from the matching FF liver and tonsil samples were done using a QiaSymphony robot in combination with the QIAsymphony DSP DNA Mini Kit and QIAsymphony RNA Mini Kit (both from QIAGEN). DNA was purified from FFPE samples using QIAamp DNA FFPE Tissue (QIAGEN) and Nucleospin FFPE DNA (Machery-Nagel) kits; RNA was purified from FFPE samples using miRNeasy FFPE (QIAGEN), Nucleospin FFPE RNA (Machery-Nagel) and ExpressArt FFPE RNAready (Amp Tec) kits. In order to test parallel purification of RNA and DNA from the same FFPE sections, the Nucleospin FFPE RNA/DNA kit (Machery-Nagel) and RecoverAll Total Nucleic Acid Isolation Kit for FFPE (Ambion) were included. Finally, in order to test automated purification procedures, DNA and RNA were purified on a QiaSymphony robot using specialized FFPE programs and the QIAsymphony DSP DNA Mini Kit and QIAsymphony RNA Mini Kit (QIAGEN), respectively. All extractions were performed in triplicate, each on three FFPE sections. Because of a limiting amount of material, extraction from breast samples with Recover All was done in duplicates only. Purifications were performed following the manufacturers' protocols with the following modification: Deparaffinisation before DNA and RNA extraction using the QIAamp FFPE DNA and ExpressArt FFPE RNAready kit, respectively, was done using QIAGEN's Deparaffinisation Solution instead of the xylene included in the kits. All DNA samples were RNase treated and all RNA samples were DNase treated during the purification. Following purification, all samples were washed on UF filter plates and eluted in QIAGEN's Elution Buffer (EB, [10 mM Tris-HCl, pH 8.5]). The fragmentation profiles of the purified RNA and DNA were estimated by on-chip electrophoresis of a 1 µL sample on an Agilent 2100 Bioanalyzer (Agilent Technologies). Since only double-stranded (ds) DNA is active in TruSeq library formation, both the total absorbance at 260 nM (NanoDropND-1000 Spectrophotometer) as well as a dsDNA specific assay (Qubit dsDNA BR Assay Kit/Qubit 2.0 Fluorometer, Invitrogen) was used to quantify the DNA concentration. The RNA concentration was determined using only the total absorbance at 260 nM.

This initial purification was followed by DNA and RNA extraction from FFPE tissues that had been routinely stored in the pathology archive for varying lengths of time after fixation. This storage time study included four tissues: tonsil (normal reactive), liver (normal non-neoplastic), bladder (carcinoma) and colon (carcinoma). Each tissue was represented with four FFPE blocks stored for 2–3, 13–15, 60–61 and 241–244 months, respectively (Table 1 and Table S1). Only the two most promising extraction kits were used: DNA Purifications were done using QIAamp DNA FFPE Tissue and Ambion RecoverAll kits. RNA Purifications were done using miRNeasy FFPE and AmpTec ExpressArt kits. All extractions were performed in duplicate using three tissue sections.

The storage time study was followed by purification of DNA and RNA from paired FF/FFPE samples of colon (19 samples), bladder (8 samples) and prostate (7 samples) carcinomas (Table 1 and Table S1). In addition, 12 FFPE colon carcinoma samples with known mutations in KRAS and BRAF were included (*the colon KRAS/BRAF variants detection set*, Table 1 and Table S1). All extractions from FF material were performed using the QiaSymphony as described above. RNA was extracted manually from FFPE blocks using the AmpTec ExpressArt kits and DNA was extracted semi-automatically from FFPE blocks on the QiaSymphony. RNA was extracted in two parallel reactions; one treated with DNase I and the other was additionally treated with Exonulcease I that catalyses the removal of nucleotides from single-stranded (ss) DNA.

### Preparation and sequencing of DNA Exome-Seq libraries

Genomic DNA libraries were prepared from FF- and FFPE-derived DNA using TruSeq DNA library preparation kits (Illumina, following the gel-free protocol) followed by exome enrichment using TruSeq exome kits (Illumina). gDNA library preparation was done according to the manufacturer's manual with the following modifications. (1) In order to avoid buffer-effects, 1.2 µg of dsDNA was processed using a QIAGEN UF filter plate, washed twice in Buffer EB ([10 mM Tris-Cl, pH 8.5], QIAGEN) and resuspended in 50 µL Buffer EB before being used as input for the TruSeq gDNA library preparation. (2) The number of PCR cycles used for amplification of FFPE-derived libraries was increased from 10 to 11. (3) After the final bead-purification step of the amplified gDNA libraries, 10 µL Buffer EB was added to the libraries, bringing the final volume up to 40 µL. The size distribution of the gDNA libraries was estimated by on-chip electrophoresis (DNA 1000 DNA chip) of a 1 µL sample on an Agilent 2100 Bioanalyzer (Agilent Technologies). The DNA concentration of the libraries was estimated using the KAPA Library Quantification Kit (Kapa Biosystems). Briefly, quantification was done using 10 µL reaction volumes containing 6 µL KAPA reagent/primer mix and 4 µL 1.000.000-fold diluted sample following the recommended procedure and the supplied standards. Quantification was carried out on three independent dilutions of each sample. Exome targeting was performed on pools of up to six gDNA libraries using 500 ng of each gDNA library according to the manufacturer's recommendations. gDNA libraries prepared from FF and FFPE samples were not pooled. After the final bead-purification step of the exome captured and amplified gDNA libraries, 30 µL Buffer EB was added to the libraries, bringing the final volume up to 60 µL. The size distributions of the captured libraries were estimated by on-chip electrophoresis (High sensitivity DNA chip) of a 1 µL sample on an Agilent 2100 Bioanalyzer (Agilent Technologies). The DNA concentration of the libraries was estimated using the KAPA Library Quantification Kit as described above. The exome captured gDNA libraries were combined into 2 nM pooled stocks, denatured and diluted to 10 pM with pre-chilled hybridization buffer and loaded into TruSeq PE v3 flowcells on an Illumina cBot followed by indexed paired-end sequencing (101+7+101 bp) on a Illumina HiSeq 2000 using TruSeq SBS Kit v3 chemistry (Illumina).

### Mutational status in BRAF and KRAS

*KRAS* and *BRAF* mutation analysis of the 11 samples in *the colon KRAS/BRAF variants detection set* were performed as described [16]. Briefly, *KRAS* codon 12, 13 and 61 and *BRAF* V600E and codon 442–474 were examined using the Lightscanner (Idaho Technology). Samples with diverging melting curves were further validated by Sanger sequencing on the 3130x Genetic Analyzer (Applied Biosystems)

### Preparation and sequencing of RNA-Seq libraries

To enable a thorough analysis of the RNA-Seq data, we required the method used for preparation of RNA-Seq libraries to provide strand-specific (directional) and paired-end information as well as facilitating multiplex sequencing. Because of the expected degradation of the RNA from the FFPE tissues, the library preparation method should not be based on selection of poly(A)+ as this would impair library preparation or at least result in a 3′ bias. Obtaining total-RNA-Seq data would furthermore facilitate a more complete view of the transcriptome. When the study was initiated in 2011, the only commercialised kit fulfilling all requirements was the Ribo-Zero technology (Epicentre, an Illumina company) for depletion of rRNA followed by library preparation using the ScriptSeq technology (Epicentre, an Illumina company). Cytoplasmic and mitochondrial rRNA were removed from total RNA using the Ribo-Zero Magnetic Gold Kit (Human/Mouse/Rat, Epicentre, an Illumina company) following the manufacturer's instructions. Briefly, rRNA Removal Solution was added to 500 ng of total RNA and incubated for 10 min at 68°C, then 15 min at room temperature. Probes with hybridized rRNA were removed by adding the RNA-probe mixture to washed Magnetic Beads followed by incubation for 5 min at room temperature, mixing by vortexing, incubation for 5 min at 50°C, magnetization and transfer of the supernatant to a RNase-free tube. The rRNA depleted RNA was purified using Agencourt RNAClean XP Kit (Beckman Coulter Inc., Brea, CA, US). Briefly, a 1.8x volume of AMPure RNAClean XP Beads was added to the rRNA depleted RNA followed by incubation for 15 min at room temperature, two 80% ethanol washes and elution into 30 µL RNase-Free water. The quality of the rRNA depleted RNA was estimated by on-chip electrophoresis (Picochip) of a 1 µL sample on an Agilent 2100 Bioanalyzer (Agilent Technologies). Savant Speed-vac (Thermo Fischer Scientific) was used to reduce the remaining sample volume to 9.5 µL followed by synthesis of directional, paired-end and indexed RNA-Seq libraries using the ScriptSeq v2 kit (Epicentre, an Illumina company) following the recommended procedure. Briefly, rRNA depleted RNA was chemically fragmented (unless severely degraded RNA from FFPE tissue was used in which case further fragmentation was omitted), following the procedure described in the appendix in the ScriptSeq manual and cDNA was synthesized from a tagged random hexamer. The cDNA was terminal tagged using a 3′-end blocked and tagged oligo followed by MiniElute (QIAGEN) purification. The di-tagged cDNA was then used as template for PCR (10 cycles for FF samples, 13 cycles for FFPE samples) using the FailSafe PCR Enzyme (Epicentre, an Illumina company), a forward primer and an indexed/barcoded reverse primer. The amplified libraries were purified using Agencourt XP Kit (Beckman Coulter). Briefly, a 1.0x volume of AMPure XP Beads (Beckman Coulter) was added to each PCR reaction followed by incubation for 15 min at room temperature, two 80% ethanol washes and elution into 20 µL RNase-Free water. The qualities of the RNA-Seq libraries were estimated by on-chip electrophoresis (High Sensitivity DNA chip) of a 1 µL sample on an Agilent 2100 Bioanalyzer (Agilent Technologies). The DNA concentration of the libraries was estimated using the KAPA Library Quantification Kit (Kapa Biosystems) following the recommended procedure and the supplied standards. The RNA-Seq libraries were combined into 2 nM pooled stocks, denatured and diluted to 10 pM with pre-chilled hybridization buffer and loaded into TruSeq PE v3 flowcells on an Illumina cBot followed by indexed paired-end sequencing (101+7+101 bp) on a Illumina HiSeq 2000 using TruSeq SBS Kit v3 chemistry (Illumina).

### Sequence analysis

The analytical workflow is described in this section and graphically presented in Figure S2. Paired de-multiplexed fastq files were generated using CASAVA software (Illumina) and initial quality control was performed using FastQC [17] or CASAVA.

Paired de-multiplexed fastq files from DNA-exome libraries were imported into the CLC Genomics Workbench (CLC Bio, version 6.0.1) running on CLC Genomics Server (CLC Bio, version 5.0.1). Adaptor sequences and bases with low quality were trimmed and reads were mapped to HG19. The *remove duplicate mapped reads* tool was used to remove paired reads that have the same start and end coordinates and are, thus, probably PCR duplicates. Variants were detected in the exome data with the CLC Bio Probabilistic Variant Caller using the following parameters: Minimum coverage  =  10; Variant probability  =  90.0; Maximum expected variants  =  4; Requirement of reads in both directions supporting the call.

Paired de-multiplexed fastq files from RNA-Seq libraries were trimmed for stretches of adapter sequences, joined into a single read if possible followed by quality trimming using commands from the CLC Assembly Cell (CLC Bio, version 4.012.84829). This resulted in three different fastq files: paired-end data, single-end data after joining of paired reads, and single-end data after elimination of one of the paired reads due to e.g. low quality. All three types of fastq files were then imported batchwise into the CLC Genomics Workbench (CLC Bio, version 6.0.1) running on a CLC Genomics Server (CLC Bio, version 5.0.1) using CLC Server Command Line Tools (CLC Bio, version 1.7.1). The imported high quality reads were mapped against gene regions and transcripts annotated by Human NCBI REFSEQ October 30, 2012 in two runs of the RNA-Seq Analysis tool of CLC Genomics Workbench. The RNA-seq tool was run with the *Use reference with annotations* option. With this option, reads are mapped against sequences corresponding to the annotated gene and transcript sequences. In the first run, only matches to the forward strand of the genes were accepted (using the strand-specific-forward option); in the second run, the reads that were not mapped in the first run were mapped allowing only matches to the reverse strand of the genes (using the strand-specific-backward option). To estimate contamination with rRNA, reads that did not map to the human transcriptome as defined by RefSeq, were mapped strand specific forward towards human rRNA (HSU13369, HSU13369, HSU13369 and NC_012920) and un-mapped reads were then mapped strand specific backward towards human rRNA. *Mapping Reports* and *Detailed Mapping Reports* were generated from each RNA-Seq analysis for each sample, exported from the CLC Genomics Workbench in Microsoft Excel format, saved as individual tab-delimited text files, and specific data were subsequently extracted and studied. For each experiment, gene-wise mapping matrices of both forward and backward RNA-Seq analyses against the human transcriptome were exported from the CLC Genomics Workbench as tab-delimited text files for exploration and statistical analysis in the R computing environment (version 3.0.1 for Windows [18]). These gene-wise mapping matrices summarize the mapping results in columns of RPKM values and counts of reads mapping to different regions, such as gene, exon, exon-exon and exon-intron. Matrices of “total exon reads” counts were selected from the gene-wise mapping matrices and analysed in the R package *Empirical analysis of Digital Gene Expression data in R* (edgeR, version 3.2.3 [19]–[23]) using the multidimensional scaling (MDS) tool for explorative analysis and the different available statistical tools for identification of differentially affected genes. The R package Hexbin was used for plotting RNA-Seq based expression profiles (base on RPKM values) by hexagonal bins. Strand specificity was estimated as the fraction of total gene reads mapping in the forward direction relative to the total number of reads mapping in both directions. Genes with fewer than 10 reads mapping in the forward direction were omitted. To estimate the fraction of duplicated sequences in RNA-Seq data post mapping (forward), BAM files were exported from the CLC Genomics Workbench and analysed using the MarkDuplicates tool in Picard tools (picard-tools-1.47,[24]).

### Accession of data

All data underlying the findings described in the manuscript are fully available without restriction. Access to the sequence data, containing person identifying information, needs signature on a controlled access form, and can be accessed at The European Genome-phenome Archive [25] using the study ID EGAS00001000737 following request to the MOMA Data Access Committee. Expression matrices are available without restriction through ArrayExpress [26] under accession E-MTAB-2523.

## Results

We studied the potential use of FFPE samples in NGS-based retrospective and prospective clinical studies, focused on detection of systematic effects of the fixation process on variant calls in DNA-Seq and on the expression profiles in RNA-Seq. The study included investigation of methods for isolation and characterization of nucleic acids purified from matching FF and FFPE specimens, preparation and quality control of exome and RNA-Seq NGS libraries, as well as analysis and interpretation of the derived NGS data.

### Isolation of nucleic acids from FFPE tissue

FFPE samples were routine diagnostic specimens, removed up to 244 months before the study start. Therefore, detailed information on the handling of samples from surgery through fixation, preparation and storage (e.g. perioperative ischemic times and fixation times) were not available. This is the typical situation for paraffin blocks from a routine pathology archive. Although pathology laboratories use standard protocols, these will vary according to the type and size of specimen, the clinical situation, the time of sampling (i.e. routine vs. out-of-hours specimens), and according to local circumstances and logistics. As a result, it will rarely be possible to estimate the expected quality of extracted nucleic acids based on the available information for a given FFPE sample. Results of the initial DNA and RNA purifications from FFPE tissues, performed in order to identify optimal extraction methods, are summarized in Table S2. In general, the method chosen for purification had a major impact on the quality and yield of RNA and DNA isolated from FFPE tissue.

#### RNA

**Figure 1:**
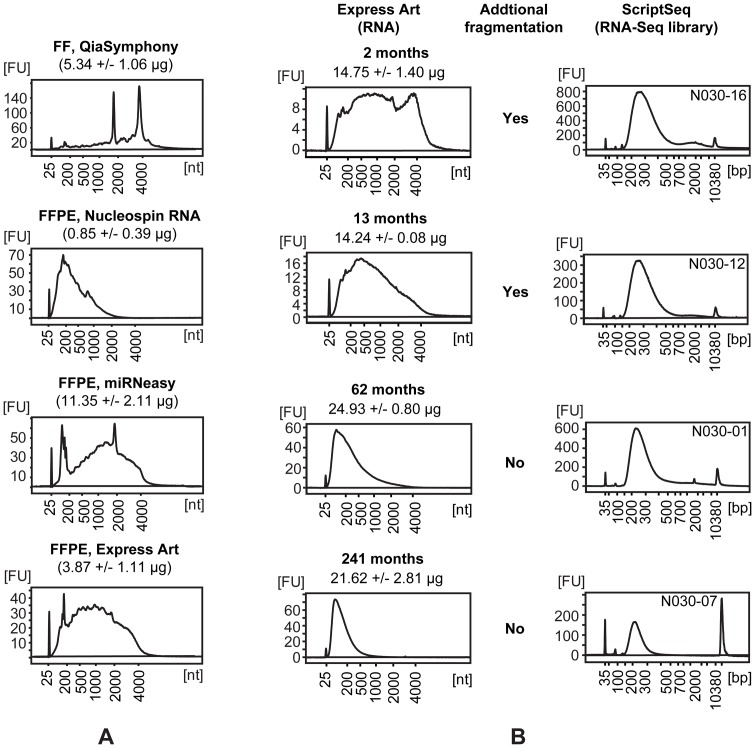
Isolation of RNA from FF and FFPE tissues and generation of RNA-Seq libraries. (A) Bioanalyzer profiles and yields of RNA isolated from FF tissue and from sections of the matching FFPE block of a normal tonsil (reactive) sample. RNA was isolated in triplicate (representative profile shown) from sections of the FFPE block using three different purification kits. Yields of RNA and purification kits are stated in the text above each profile. (B) Bioanalyzer profiles of RNA (left) isolated by ExpressArt FFPE RNAready (Amp Tec) from FFPE samples of normal tonsil (reactive) stored for different times as indicated above the profile together with the yields. Isolations were conducted in triplicate and a representative profile is shown. The resulting profiles of RNA-Seq libraries (right, with library ID top-right), with or without preceding fragmentation of the RNA as indicated in the middle section.

miRNeasy FFPE (QIAGEN) and ExpressArt FFPE RNAready (Amp Tec) kits resulted in higher yields and less severely degraded RNA as compared with Nucleospin FFPE RNA (Machery-Nagel) (Figure 1A, Table S2). Therefore, the first two kits were used for subsequent RNA purifications from *the storage time FFPE set*. Only the ExpressArt FFPE RNAready kit was used for RNA purifications from the three paired FF/FFPE sets. Sufficient yields (> 500 ng total RNA) for preparation of RNA-Seq libraries were obtained from all samples. Storage time of the FFPE blocks was found to have a negative impact on RNA quality, but not on the yield (Figure 1B, Table S2). However, relatively intact RNA could be isolated within the first year of storage.

#### DNA

DNA isolated from FFPE tissue was found to be relatively intact, although there was a tendency for degradation to increase with storage time. As dsDNA, in contrast to ssDNA, can be ligated to adapters during the preparation of gDNA-Seq libraries for NGS, it is important to measure the concentration of dsDNA using a suitable method and not to rely on A260 nm measurements, which does not distinguish between dsDNA and ssDNA. The QIAamp DNA FFPE Tissue (QIAGEN) gave higher dsDNA yields than the Nucleospin FFPE DNA kit (Machery-Nagel) (Table S2).

Parallel purification of RNA and DNA from the same FFPE sections was tested using the Nucleospin FFPE RNA/DNA kit (Machery-Nagel) and RecoverAll Total Nucleic Acid Isolation Kit for FFPE (Ambion). However, this resulted in low yields of both RNA and DNA and these purification methods were not further included.

### Preparation and sequencing of DNA Exome-Seq libraries

Preparation of DNA Exome-Seq libraries was successful for ten of the 19 samples in *the paired FF/FFPE colon set* and for eight of the 11 samples of *the colon KRAS/BRAF variants detection set*, but failed for the remaining 43 FFPE samples (29.5% (18/61) success rate). The Illumina TruSeq Exome-sequencing libraries were obtained by preparation of whole genomic sequencing (gDNA-Seq) libraries followed by capture of the exome target on biotinylated probes, complementary to the exome and UTR regions. A total of 500 ng of gDNA-Seq library was required for the subsequent capture reaction and we found that the major challenge in preparing exome-sequencing libraries from FFPE-derived DNA was to obtain a sufficient amount of the gDNA-Seq libraries. We observed that degraded DNA invariably produced very low levels of gDNA-Seq library but it was not caused by the shorter DNA fragments per se. Thus, the degree of degradation must correlate with another FFPE-related DNA modification that prevents efficient gDNA-Seq library production.

In agreement with the notion that DNA is degraded over time during FFPE-storage, we found that gDNA-Seq library yields were inversely correlated with the storage time of the FFPE blocks, i.e. the longer an FFPE block had been stored, the lower the gDNA-Seq library yield. Interestingly, a comparison of the use of DNA from FFPE-blocks fixed and then stored for no longer than three months with DNA from control FF samples for library preparation, showed a significant reduction in efficiency, even in these freshly prepared FFPE-blocks. Therefore, in addition to the negative correlation between FFPE block storage time (degradation) and gDNA-Seq library preparation efficiency; there is a general effect of tissue fixation and paraffin embedment that reduces the ability to produce gDNA-Seq libraries from FFPE-derived DNA.

During gDNA-Seq library preparation, DNA fragmentation was followed by three enzymatic steps resulting in the addition of a total of 120 bp of adapter sequence. After adapter ligation, gDNA-Seq libraries were PCR amplified. Comparison of the size distribution of the DNA fragments prior to adapter ligation with the unamplified gDNA-Seq library after adapter ligation showed that the FFPE-derived DNA was performing well in these steps of the gDNA-Seq library preparation. This indicated that it was the final PCR amplification step in the library preparation that was compromised when using FFPE-derived DNA as the starting material. This was probably a result of DNA modifications caused by formalin fixation of the tissue. In order to increase the number of successful gDNA-Seq libraries from FFPE-DNA, the number of PCR cycles was increased from 10 to 11.

If less than 1 µg of dsDNA was used for gDNA library prep, the complexity of the resulting gDNA and exome library was reduced. This was observed as an increase in the fraction of PCR duplicates in the data. Since only a fraction of FFPE-derived DNA can be amplified during gDNA library prep, this effectively corresponded to an input of less than 1 µg of dsDNA. In agreement with this, we found that the PCR duplication rate was significantly increased in FFPE samples (by 60% – 85%) compared to the FF control samples (approximately 30%), as measured by CASAVA during library QC. The relatively high level of duplicates in the control FF samples was a result of the increase in the number of PCR cycles. The recommendation for TruSeq exomes was to sequence three samples in each flow cell lane. Importantly, sequencing of the libraries after exome targeting showed that the loss in reads due to PCR duplicates in FFPE samples could largely be overcome by sequencing fewer samples in each lane. We found that the sequencing of three FFPE exomes across two lanes resulted in a number of unique reads comparable to that found for the libraries prepared from matching FF-sample DNA or from high quality control DNA sequenced as three samples per lane. Exome capture and sequencing of gDNA-Seq libraries that produced significantly less than 500 ng of gDNA-Seq library resulted in an increase in PCR duplicates to a level where deeper sequencing may be needed to produce meaningful data. This corresponded to an exclusion of all heavily degraded FFPE-derived DNA from exome sequencing using the protocol described here.

Neither increasing the amount of dsDNA used as input for gDNA-Seq library preparation from 500 ng to 2 µg nor parallel preparation of two gDNA-Seq libraries followed by pooling before exome capture reduced PCR duplicate level. Because cytosine deamination to uracil is a common form of damage in FFPE-derived DNA, we tested the use of the KAPA HiFi Uracil+ DNA Polymerase (Kapa Biosystems) for library amplification. However, the resulting libraries had a higher PCR duplication level than the libraries prepared using the DNA polymerase provided in the TruSeq DNA library preparation kits.

### Analysis of DNA Exome-Seq data

**Figure 2:**
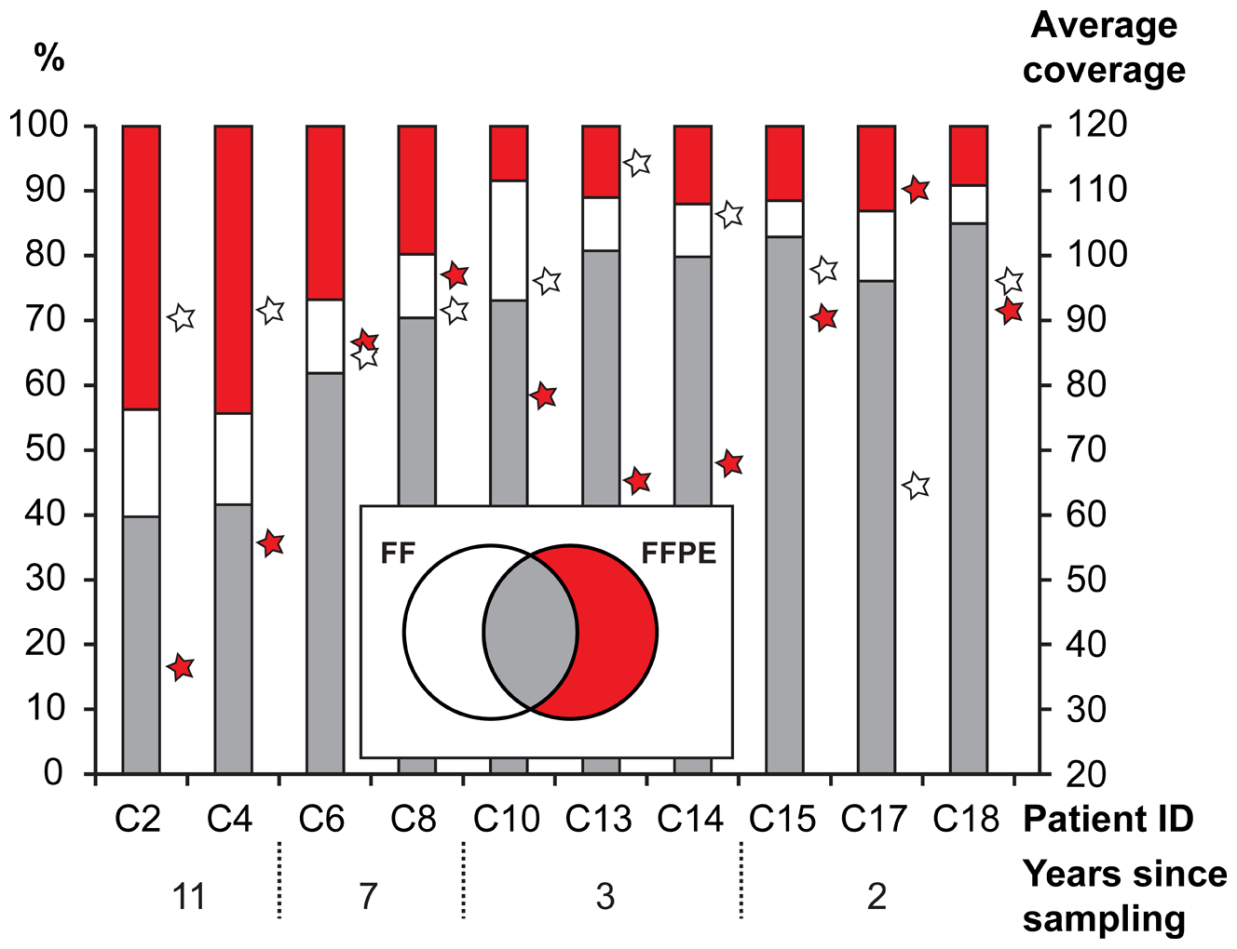
Single nucleotide variants detected in DNA-Exome-Seq data from the paired FF/FFPE samples. The percentage of common (grey), exclusively FF (white) and exclusively FFPE (red) single nucleotide variants are shown as bars referring to the left axis. The average coverage is shown as stars for FF (white) and FFPE (red) on the right axis. Patient ID and number of years of storage are shown below.

Exome data from ten FF/FFPE pairs from *the paired FF/FFPE colon set* were available for further analysis. The number of total reads, number of mapped reads, percentage of reads mapped, percentage of duplicates and number of variants called are listed in Table S3. In general, more reads were generated for the FFPE samples to compensate for the higher duplication rate. Insert sizes were shorter for the FFPE samples, both for all samples in general and for the oldest FFPE samples in particular (Figure S3). Furthermore, because of the smaller insert size, FFPE samples contained more adaptor sequences leading to more bases being trimmed. There was no effect of storage time on the insert size of the FF samples. The percentage of reads mapping to the genome was higher for FF compared with FFPE samples. In contrast, the percentage of duplicates was higher for FFPE samples, especially in the older samples. To assess the effect of the FFPE treatment on the DNA Exome-Seq reads we compared the (1) mapping, (2) error and (3) detected variant characteristics for the ten pairs of matched FF and FFPE samples, subject to four different storage times. There was a tendency for FFPE samples to exhibit a smaller fraction of mapped reads than observed for FF samples, and for the fraction to decrease with increasing storage time (Figure S4A). Specifically, we observed a larger fraction of non-perfectly mapped reads, as well as reads mapped with unaligned ends, in FFPE compared to the FF samples (Figure S4B). Moreover, the older the samples, the more pronounced the difference between the FFPE and FF samples. An examination of the actual differences between the reads and the reference at the mismatch positions (Figure S4C) indicated a systematic pattern in the types of errors that accumulate over time, with an excess of T<->C and A<->G differences. In the comparison of the variants detected in the samples, there appeared to be little difference in the types detected for younger samples; for older samples there was a tendency for a smaller proportion of T -> C and A -> G variants in the FFPE relative to the FF samples (Figure S4D). Comparing the identity of the detected variants in the FF and FFPE data within the paired FF/FFPE samples, revealed that 70% – 80% of the variants were pairwise in common for samples stored less than three years. For samples stored longer than three years, the fraction of variants detected exclusively in data from FFPE samples was increased while the fraction of FF exclusive variants was independent of storage time. Relative fractions of variants and average coverage are presented in Figure 2 and absolute numbers in Figure S5.

### The *KRAS/BRAF* test

As a proof-of-principle that exome sequencing of FFPE samples can be used to identify actionable variants, 11 samples with known *KRAS* and/or *BRAF* mutations were selected for exome sequencing. Eight of these were successfully sequenced and all previously identified *KRAS/BRAF* mutations were also identified by exome sequencing with a frequency between 6 – 46% (Table 2). In addition, *KRAS* hotspots mutations were found in two samples previously tested negative for *KRAS* mutations by Sanger sequencing. Both had a frequency < 2% indicating an increased sensitivity achieved by NGS. However, since FF tissue were not available for these samples this has not been validated further.

**Table 2:** Variants identified in the colon KRAS/BRAF variants detection set.

	KRAS			BRAF	
Sample	C.34G>A, het(P.Gly12Ser)	C.34G>T, het(P.Gly12Cys)	C.59C>T, het(P.Thr20Met)	C.1799T>A, het (P.Val600Glu)	C.1432+10T>C
C20	0	0	0	29.13 #	28.36 #
C21	0	0	1.5 ∧	23.64 #	0
C23	0	0	24.24 #	32.43 #	0
C25	0	0	0	28.36 #	0
C27	0	1.12 ∧	0	6.06 #	0
C28	0	0	0	45.75 *	0
C29	0	14.63 #	0	0	0
C30	45.58 #	1.37	0	0	0

#: Mutations previously identified with Sanger sequencing.

*: Mutations not tested with Sanger sequencing.

∧: Mutation NOT previously identified by Sanger sequencing.

### Preparation and sequencing of RNA-Seq libraries

Notably, it was possible to prepare and sequence RNA-Seq libraries from all samples tested, even when the RNA was isolated from specimens sampled, fixed and embedded two decades previously (Figure 1B). Size profiles of the isolated RNA range from relatively intact RNA from fresh fixed samples to highly degraded RNA from older samples. These differences are a challenge when preparing RNA-Seq libraries, since it is preferable that the size distributions of the obtained libraries are relatively equal. Substantial differences in size distribution and too large insert sizes may affect the final transcript coverage. Therefore, we tested differential chemical degradation of RNA in order to obtain similar RNA size profiles before library preparation. By varying incubation temperature and time, and the working concentration of the fragmentation reagent, it was possible to achieve differential degradation of intact, high quality RNA. Unfortunately, it was not possible to obtain reproducible results when applying differential degradation to the partially degraded RNAs isolated from FFPE tissue samples. This was probably caused by difficulties in predicting the optimal settings from a size profile of degraded RNA, leading to sub-optimal fragmentation. Therefore, we suggest applying fragmentation (such as the method included in the Ribo-Zero kit) to partially degraded RNA and omitting further fragmentation if the RNA is already degraded below 200 nt. Figure 1B shows examples of RNA profiles and resulting RNA-Seq library profiles. For depletion of rRNA, we applied the probe-based technology implemented in the Ribo-Zero kit from Epicentre. However, as described below, we experienced some variation in the success of rRNA depletion between libraries and tissues. It should be noted that both fragments of rRNA as well as the capture probes from the Ribo-Zero kits will function as templates in the ScriptSeq reaction. Nonetheless, when applying a strand-specific library preparation method, the reads mapping to rRNA can be distinguished during analysis, since the sequences originating from rRNA fragments will map to the sense strand, while sequences from the rRNA capture probes will map to the anti-sense strand. This can assist in troubleshooting incidences of higher than expected rRNA reads in the RNA-Seq libraries. One of the challenges in the Ribo-Zero method is to achieve sufficient mixing when adding the removal reaction (mixture of RNA and rRNA probes) to the washed, resuspended beads. Insufficient mixing will lead to the formation of vesicles and an excessive number of rRNA reads in the RNA-Seq library. In addition, freezing of the magnetic rRNA probe binding beads will result in decreased capture of the rRNA probes. The prepared RNA-Seq libraries were multiplexed paired-end sequenced (101+7+101 bp) on an Illumina HiSeq 2000.

### Pre-mapping analysis of RNA-Seq data

Contaminating ssDNA in RNA isolated from FFPE tissues would result in sequence reads mapping to both strands with a resultant reduction in strand-specificity. To study this, we repeated RNA-Seq library preparations in six of the FFPE colon carcinoma samples, using RNA treated with DNase and Exonuclease I to remove both dsDNA and ssDNA. Using multi-dimensional scaling of the Total Exon Reads of the RNA-Seq forward data, we found the paired samples to group together (Figure S6A) and the RPKM values of the paired data to be highly correlated (Figure S6B). We did not observe any significant effect of DNase treatment on either the distribution, or the mean of the strand-specificity for each of the six paired samples (Figure S6C). Thus, contamination with ssDNA does not appear to be a major problem when isolating RNA from FFPE samples. The RNA-seq data from the six paired DNase treated samples were consequently combined pairwise and re-mapped to represent the six individual FFPE samples.

The de-multiplexed paired-end fastq files from the RNA-Seq libraries were trimmed for stretches of adapter sequences, joined into a single read if possible, and were then subjected to quality trimming using commands from the CLC Assembly Cell. In the colon and prostate sets, we generally observed that less than 1.5% of reads were removed due to adapter content, with a higher fraction of reads removed in FFPE compared with FF libraries (Figure S7A). In the bladder set, less than 0.4% of reads in the FF libraries were removed due to adapter content compared with less than 0.1% in the FFPE libraries (Figure S7A). All but seven libraries from *the storage time FFPE set* had low adapter contents (Figure S8A). Adapter trimming affects both adapter dimers and library elements with very small inserts. The slightly higher fraction of adapter trimmed reads in the FFPE compared with the FF libraries was probably the result of the shorter insert sizes in FFPE libraries, resulting in a higher fraction of paired reads which could be joined into a singlet in the FFPE compared with the FF libraries (Figure S7B). In *the storage time FFPE set*, we observed a high fraction of joined reads across sample ages (Figure S8B). The pre-mapping procedures resulted in three different fastq files per sample: paired-end data, single-end data after joining of paired reads, and single-end data after elimination of one of the paired reads due to e.g. low quality. The majority of the reads were joined reads, with a higher fraction in FFPE compared with FF libraries (Figure S7C). In *the storage time FFPE set*, a majority of the reads were found to be joined reads (Figure S8C).

### Post-mapping analysis of RNA-Seq data

**Figure 3:**
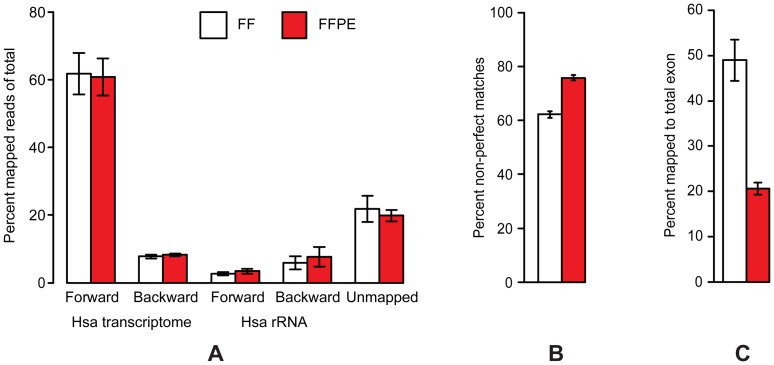
Post mapping results from RNA-Seq of *the paired FF/FFPE colon set*. (A) The fractions of reads mapping to the different targets. (B) Fractions of non-perfect matches among matches in RNA-Seq forward. (C) Fractions of reads mapping to total exon regions (total intron is the remaining fraction up to 100%). Data from FF and FFPE specimens are shown as white bars and red bars, respectively.

The trimmed reads were mapped against gene regions and transcripts as annotated by Human NCBI REFSEQ (October 30, 2012) using the RNA-Seq analysis tool of the CLC Genomics Workbench. The reads were sequentially mapped strand specific against the forward and then backward human gene regions and transcripts. The reads that did not map to the human transcriptome were mapped sequentially strand specific forward and then backward towards Human rRNA. The proportions of reads mapping to the different targets are presented in Figure 3A (*the colon set*), Figure S9A (*the bladder and prostate sets*) and in Figure S10A (*the storage time FFPE set*). In *the colon set*, essentially no differences were observed between FF and FFPE libraries in the fraction of reads mapping to the different target groups. A total of 61% of reads mapped to the human transcriptome in the forward orientation and 8% in the backward orientation, while 3% mapped to human rRNA in the forward orientation and 7% in the backward orientation, leaving 21% un-mapped. In *the prostate and bladder sets* (Figure S9A), we observed a shift in the distribution for the FFPE samples as a higher fraction of reads was found either to map to rRNA (especially backward in *the bladder set* indicating a technical issue with insufficient capture of the rRNA probes) or not to be mapped (in *the prostate set*). In *the storage time FFPE set* (Figure S10A), we observed some heterogeneity in distribution between the different samples and storage times, but in general a majority of reads were found to map to the human transcriptome in the forward orientation and no effects of storage time of samples were observed.

More than 92% of the reads mapping to the human transcriptome were found to map uniquely in both FF and FFPE libraries in all sets. However, the fractions of non-perfect matches were higher in FFPE compared with FF data (Figure 3B and Figure S9B). In *the storage time set* there was a tendency to see an increasing fraction of non-perfect matches as a function of increasing storage time (Figure S10B). This is probably due to the chemical modifications introduced by the fixation process. Based on this increased error rate in RNA-Seq data from FFPE tissue, we recommend applying longer sequences (e.g. 101 bp) to FFPE libraries to ensure unique matches.

We did not observe any significant differences in strand specificity between RNA-Seq libraries from FF or FFPE samples (data not shown). The complexities of the libraries were estimated by the post mapping fraction of duplicated sequences and are presented in Figure S11. In general, the duplication rate was found to vary from sample to sample and from set to set. However, higher duplication rates were observed in FFPE relative to FF libraries. In *the storage time set*, increased storage time of the FFPE samples was correlated to increased duplication rate, except for three sets of paired FF/FFPE samples. When assessing the proportion of reads mapping to exon and intron regions of the annotated transcriptome, RNA-Seq data from FF samples were found to have an almost equal proportion while RNA-Seq data from FFPE samples were skewed towards a much lower proportion of exonic reads (Figure 3C; Figure S9C and Figure S10C).

**Figure 4:**
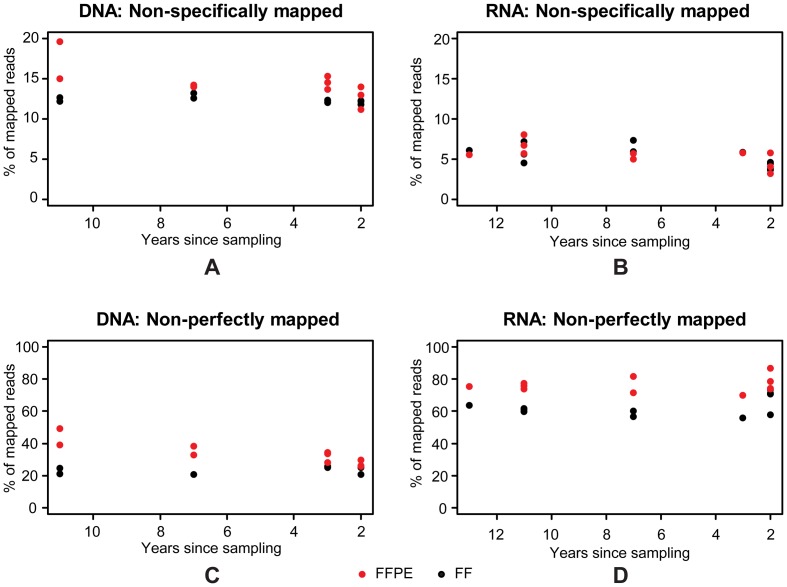
Effects of storage time on post-mapping results from the paired FF/FFPE samples. Fractions of non-specifically mapped DNA-Exome-Seq (A) and RNA-Seq (B) reads; fractions of non-perfectly mapped DNA-Exome-Seq (C) and RNA-Seq (D) reads for FF (black) and FFPE (red) for each of the ten samples by number of years since sampling.

To summarize the general tendencies in mapping of FF and FFPE NGS data (using paired-end 2x 101 bp sequencing) from DNA and RNA, we found: (1) A higher fraction of non-specifically mapped reads was observed in data from DNA compared with RNA. (2) There tended to be a higher fraction of non-specifically mapped reads in data from FFPE-derived DNA, while no differences were found between FF and FFPE data in RNA (Figure 4A and B). (3) There was a tendency for a higher fraction of non-perfectly mapped reads in FFPE compared with FF data from both DNA and RNA; and the fraction of non-perfectly mapped reads was in general higher for RNA compared with DNA (Figure 4C and D). (4) In NGS data from FFPE-derived DNA there tended to be an increased fraction of both non-specifically and non-perfectly mapped reads as a function of increasing storage time, while no effects of storage time were observed in FF samples or in RNA data (Figure 4).

### The FFPE process affects the expression profiles

**Figure 5:**
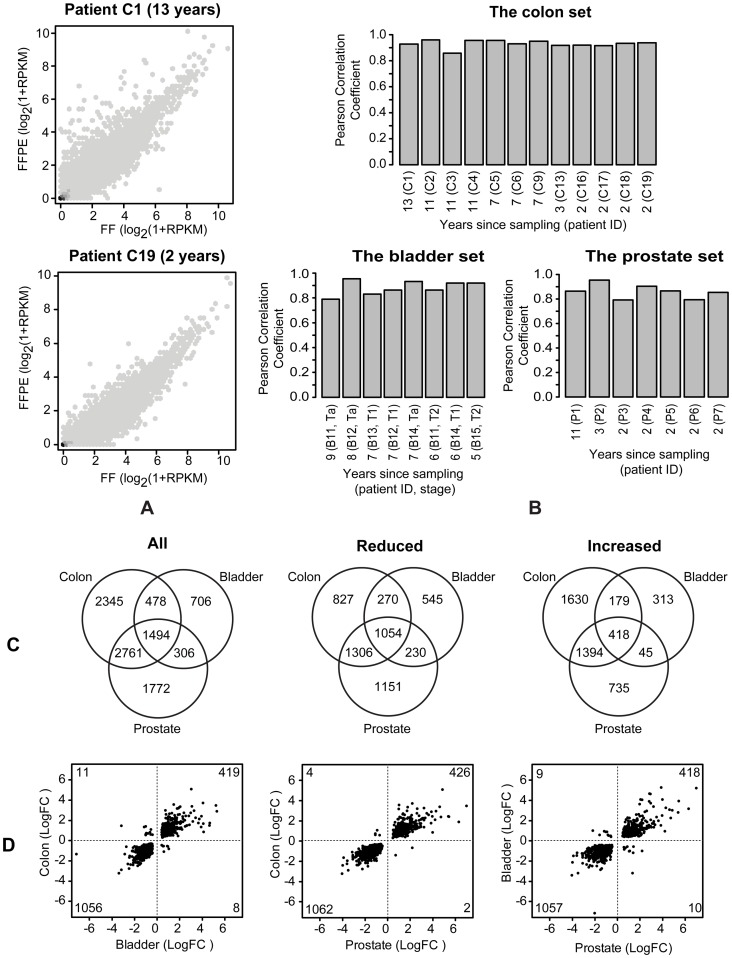
Effect of the FFPE process on the expression profiles. (A) Correlation plots of log_2_(1+RPKM) values for FF and FFPE colon samples stored for 13 years (top) and 2 years (bottom). (B) Pearson correlation coefficients of log_2_(1+RPKM) values for FF vs. FFPE from the three paired FF/FFPE sample sets. (C) Intersection analyses of the results from paired statistical tests for significantly (FDR<0.05) affected genes comparing FF and FFPE samples within each sample set (divided into all affected, reduced and increased genes). (D) Plots of log-fold change values of the 1,494 genes affected in common, among all affected genes, for each pairwise combination of the three tissue sets with numbers of genes indicated for each quadrant.

To investigate the effects of the FFPE process (fixation, processing, embedding and storage) on expression profiles, expression values from the paired FF/FFPE samples (RPKM of exonic reads) were plotted and the Pearson Correlations Coefficient was calculated for each set. We found the expression profiles obtained from FF and FFPE samples to be highly correlated, both in the recently collected samples and, importantly, even in samples collected 14 years previously (Figure 5A). Analysis of the Pearson Correlations Coefficients from all FF/FFPE pairs (Figure 5B), revealed a high degree of correlation (0.90 +/- 0.05) across storage time and tissue type. To further investigate the effects of the FFPE procedure, we conducted statistical tests for significantly affected genes between matched pairs of FF and FFPE samples in the three tissue sets (colon, bladder and prostate). MDS analysis (Figure S12A, B and C) showed a separation of the FF and FFPE samples; the FFPE samples were a quite heterogeneous group while the FF samples tended to cluster more closely together, especially in *the prostate set*. The paired study design was evident for some pairs which clustered closely together in MDS, while other pairs were more distantly located. It should be recalled that the FF and FFPE samples are indeed sampled from two different tissue locations. As a result, tissue heterogeneity (including differences in e.g. the percentage of tumour cells, in the sub-clonal composition, and in the tumour microenvironment) can be expected to result in variations in the profiling results comparing paired FF and FFPE samples. Paired statistical tests for significantly (FDR<0.05) affected genes in FF and FFPE sample profiles revealed 7,078 genes to be affected in *the colon set* (3,457 genes with reduced expression and 3,621 genes with increased expression in FFPE relative to FF, 19334 genes tested in total), 2,984 affected genes in *the bladder set* (2,029 reduced and 955 increased, 19346 genes tested in total) and 6,333 genes affected in *the prostate set* (3,741 reduced and 2,592 increased, 19410 genes tested in total). Ratio-intensity (MA) plots from the three tests with the significantly affected genes highlighted are presented in Figure S12D, E and F. The results of intersection analyses of the three tissue sets of affected genes (divided into all affected, reduced and increased genes) are presented as Venn diagrams in Figure 5C and showed 1,494, 1,054 and 418 genes to be commonly affected among all affected, reduced and increased genes, respectively. Gene ID and test statistics of the 1,494 genes are listed in Table S4. Plotting the log-fold change values of the 1,494 genes for each pairwise combination of the three tissue sets (Figure 5D) showed that the direction of the expression change was shared among the three sets for the vast majority of the affected genes. Furthermore, a larger fraction (71%) of the commonly affected genes was found to have reduced expression in FFPE compared with FF samples (Figure 5D).

### Identification of differentially expressed genes in *the colon set*

**Figure 6:**
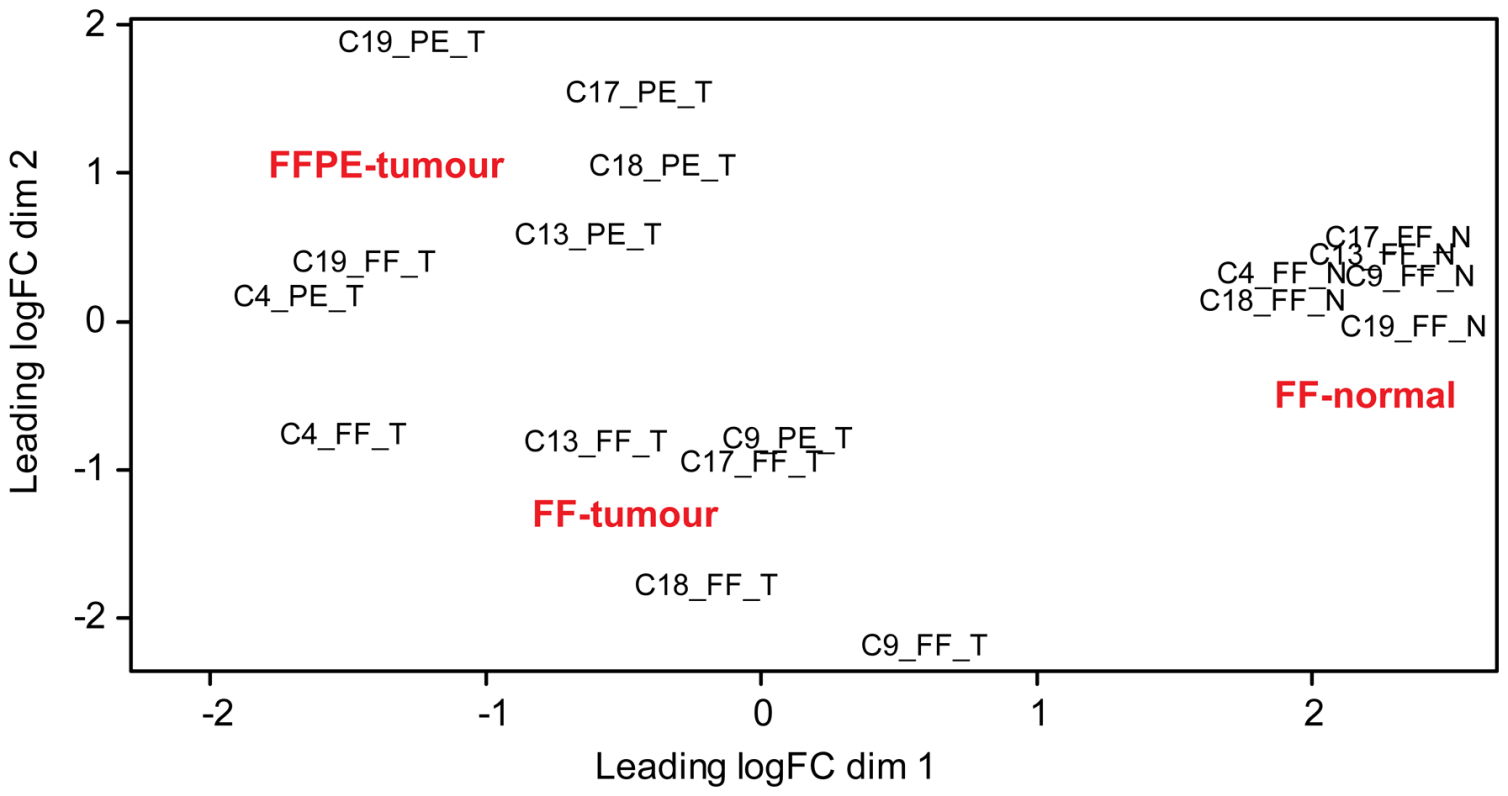
MDS analysis of expression profiles of the colon trio-sets. MDS analysis of the expression profiles of the six trio-sets (FF and FFPE samples from colon tumours with adjacent normal colon tissue) from *the paired FF/FFPE colon set*. CX: patient ID; T: tumour; N: normal; FF: fresh frozen; PE: FFPE.

The sequenced fraction of *the colon set* consisted of 12 paired FF/FFPE colon tumour samples, of which six had an additional sample of adjacent normal colon tissue. These six trio-sets of tumour and normal tissue were used to study the concordance between the expression profiles of affected genes in tumour versus normal tissue (both FF) and the profile deduced from the FFPE tumour samples. MDS analysis of the expression profiles of the trio-sets (Figure 6) revealed a clear separation of the FF normal samples from the tumour samples in dimension 1 (horizontal) and a separation of the FF and FFPE tumour samples, with a single exception, in dimension 2 (vertical). The FF and FFPE samples of identical tumours were found to be located roughly on the same horizontal axis but clearly separated from each other vertically confirming (1) the paired nature of the tumour samples, and (2) differences in the expression profile between paired FF and FFPE samples. However, since the FF and FFPE samples are sampled at two different locations in the tumour, any sampling effects and FFPE effects are confounded. MDS analysis of the expression profiles of the FF tumour and FF normal samples (Figure S13A) revealed (1) clear separation in dimension 1 (horizontal) and (2) that the FF normal samples formed a homogenous cluster while the FF tumour samples were more heterogeneous. Paired statistical tests for significantly (FDR<0.05) affected genes between tumour and normal samples (both FF) revealed 3,487 genes to be affected (1,471 genes with increased expression and 2,016 genes with decreased expression in tumour, relative to normal colon tissue). A ratio-intensity (MA) plot from the test with the significantly affected genes highlighted is presented in Figure S13B. Since the specimens in *the colon set* included both benign and malignant tumours (see Table S1) it is possible to compare the genes affected in these sample types within the FF and FFPE sets. MDS analysis of the expression profiles of the 12 paired FF/FFPE samples revealed a clear separation of the 12 FFPE samples into malignant and benign groups (Figure S14A). The 12 FF samples were also found to separate into malignant and benign groups, but with a larger variation in the malignant group (Figure S14B). Thus, the MDS analyses justify testing for affected genes comparing benign and malignant samples within the FF and FFPE sets. Paired statistical tests for significantly (FDR<0.05) affected genes comparing benign and malignant samples, revealed 471 and 1,207 genes to be affected in the FF and FFPE sets, respectively. Ratio-intensity (MA) plot from the two tests with the significantly affected genes highlighted are presented in Figure S14C and D. A Venn diagram of the two sets of affected genes revealed 158 genes to be in common (Figure S14E). A total of 66 of the 1,207 genes in the FFPE set and 28 of the 471 genes in the FF set were found among the 1,494 genes significantly affected between FF and FFPE samples.

### Identification of differentially expressed genes in the bladder set

**Figure 7:**
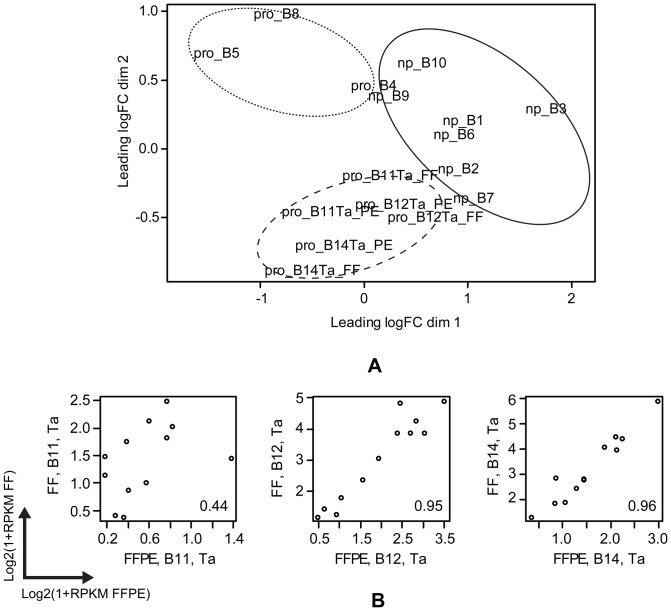
MDS analysis and pairwise plots of the expression profiles from bladder specimens. (A) MDS analysis of the ten FF bladder carcinoma samples and the three FF/FFPE pairs of Ta tumours based on the expression profiles of the genes included in the previously published [27] 12-gene expression signature for progression in bladder carcinoma. Pro: progressing carcinoma; np: non-progressing carcinoma; FF: fresh frozen; PE: FFPE. (B) Pairwise plots of log_2_(1+RPKM) values from the three FF/FFPE paired samples with Pearson correlation coefficients noted in the bottom-right corner.

The two bladder carcinoma sample-sets included in this study, *the bladder set* of eight paired FF/FFPE and *the FF bladder signature conservation set* of ten FF samples of bladder carcinoma, were selected to investigate if, as a proof-of-principle, the 12-gene expression signature for progression in bladder cancer [27] can be found in FFPE samples. The ten FF samples were selected among the original samples previously used to define the expression signatures for progression [28], in order to confirm that the signature could be transferred from microarray to the NGS platform. The ten samples are all Ta tumours collected from individual patients of which three show later progression. Six of the eight paired FF/FFPE samples comprise pairs of Ta tumour (initial) and later progressing tumour samples from three patients. MDS analysis of the ten FF bladder carcinoma samples and the three FF/FFPE pairs of Ta tumours based on the expression profiles of the genes included in the 12-gene expression signature for bladder carcinoma progression (BIRC5, CDC25B, COL18A1, COL4A1, COL4A3BP, FABP4, KPNA2, MBNL2, MSN, NEK1, SKAP2 and UBE2C) [27], resulted in separation of the recurrent bladder carcinoma samples from the progressing bladder carcinoma samples in dimension 1 (horizontal) and a separation of the progressing bladder carcinoma samples in dimension 2 (vertical) (Figure 7A). Some heterogeneity was observed among the progressing Ta tumours, since the three FF/FFPE pairs of Ta tumours were separated from the three progressing Ta tumours of the FF-set. Notably, the FF/FFPE pairs were found to be closely linked and their paired nature was especially evident for the patient samples B12 and B14. In contrast, the FF and FFPE samples from patient B11 were more distantly located. A similar pattern was also clearly shown by plotting the RPKM values from the three FF/FFPE paired samples (Figure 7B). Analysis results from the FF and FFPE samples of patients B12 and B14 were highly correlated while FF and FFPE samples from patient B11 were less clearly correlated. The bladder signature gene *KPNA2* was among the 1,494 genes found to be significantly affected between FF and FFPE samples.

## Discussion

We performed a systematic study defining key parameters for successfully applying NGS in the analysis of FFPE tissues. The study results have important implications. The ability to carry out molecular analyses of DNA and RNA in routine diagnostic FFPE tissues that had been fixed, prepared and stored for up to two decades prior to the study, provides further proof of the robustness of NGS technology. This in turn emphasizes the potential role of this technology in opening up paraffin block archives to high-throughput sequencing for use in both retrospective and prospective clinical studies.

We evaluated available commercial FFPE extraction kits for their suitability for the purification of both RNA and DNA from different tissue types and after storage times after fixation. Extracted DNA and RNA from matching FF and FFPE specimens were used for the preparation of targeted genomic and whole transcriptome sequencing libraries which were sequenced and the resulting data analysed.

When analysing the results of our studies on paired FF/FFPE samples, it is important to remember that these were sampled from different tissue locations, albeit within the same tumour. Thus, tumour heterogeneity (e.g. in the form of varying numbers of tumour cells, different tumour subclones, tumour necrosis, and vascular and other differences in the tumour microenvironment) will generate a background of variation, against which changes in NGS data derived from the paired FF and FFPE samples has to be analysed. In spite of these challenges, we were able to identify clear and close correlations between the expression profiles in RNA-Seq data, and detect similar sequence variants in DNA Exome-Seq data for the FF/FFPE pairs. Thus, in spite of the potential problems of both sampling variation and tumour heterogeneity, our study shows that NGS-based analysis of FFPE samples may provide valid, robust data.

It will rarely be possible to estimate the expected quality of extracted nucleic acids based on the available information for a given FFPE block. Instead, standard methods of purification from FFPE tissue should be adopted and the extracted nucleic acids should be validated to decide if the material is of sufficient quality and quantity to be used for NGS based studies. The different FFPE specimen extraction methods tested varied in both quality and yield of nucleic acids. However, we were able to identify a purification method which, in our hands, was the best among those tested in terms of yield, degradation and (for DNA) fraction of dsDNA. For RNA extraction, our results are in agreement with Boeckx *et al.* who reported that the RNeasy FFPE Isolation Kit (QIAGEN) performed well and was superior to the NucleoSpin FFPE RNA Isolation Kit (Macherey-Nagel) [29]. Most of the published studies that included isolation of FFPE specimen nucleic acids did not test different isolation strategies. This may partly explain some of the variation in the results reported. It is evident from both academic and commercial publications that new nucleic acid extraction methods for FFPE specimens are under continued development. Future studies should evaluate these systematically, especially with regard to their ability to counteract the detrimental effects of fixation, tissue block preparation and storage.

Preparation of DNA Exome-Seq libraries was successful for ten samples from *the paired FF/FFPE colon set* and for eight samples of *the colon KRAS/BRAF variants detection set* but failed for the remaining 43 specimens (29.5% (18/61) success rate). The main reason for the failure was non-efficient amplification of the libraries following adaptor ligation, most probably as a result of DNA modifications caused by the fixation and subsequent storage. This underlines the need for improved extraction strategies that are more efficient in reversing these modifications. Since cytosine deamination to uracil is one common type of damage in FFPE-derived DNA, we tested an alternative uracil tolerant DNA polymerase. However, this showed no advantage over standard DNA polymerase. Nonetheless, we believe this may be a fruitful area to explore, and additional DNA polymerases should be tested in the future for their ability to facilitate more efficient amplification of libraries. An alternative approach may be pre-treatment of FFPE-derived DNA with uracil-DNA glycosylase as reported by Do *et al.* [30], [31].

We and others [1], [5], found that sequencing of FFPE-derived DNA resulted in higher duplication rates, smaller insert sizes, a lower fraction of mappable reads, a larger fraction of non-perfectly mapped reads and reads mapping with unaligned ends. Schweiger *et al.* reported that storage time of FFPE blocks had only a minor influence on sequencing quality [1]. In contrast, we found that storage time was correlated to a decreased fraction of mapped reads, an increased fraction of non-perfectly mapped reads and reads mapping with unaligned ends, and an increased fraction of variants detected exclusively in FFPE samples. It should be stressed, that the conclusions from both our study and that of Schweiger *et al.* [1] are based on a low number of samples stored for longer periods. Additional work is needed to clarify the effects of FFPE block storage time on the sequencing quality. Fixation induced noise can be reduced by increased coverage as achieved by targeted sequencing [3]. This also facilitates the accurate detection of variants in specific genes, as shown in this and in previous [4], [5] studies. In spite of the relatively low number of specimens included in this and in previous studies, NGS seems to be a promising technology for the analysis of variant frequencies in FFPE tissue derived DNA. Our findings add to previous evidence that together highlight the increased sensitivity achieved using NGS to detect low frequency variants. However, it still remains an open question whether such low frequency variants are of clinical relevance.

In contrast to the challenges we encountered when applying NGS to FFPE-derived DNA, we were surprised to be able to prepare functional RNA-Seq libraries from all 56 FFPE samples we tested, including even samples that had been routinely collected and fixed two decades previously, and then stored at room temperature in the routine pathology archive. Our results add to the previously reported studies, which show successful application of NGS for molecular profiling of a range of samples, differing widely in terms of tissue types and storage times. Using sequentially mapping of strand specific RNA-Seq data we found no major differences between FF and FFPE libraries in the fractions of reads mapping to the different targets. However, we did observe some variation between the different tissue sets, mainly caused by an increased fraction of rRNA reads. We found the Ribo-Zero technology for depletion of rRNA to perform well on degraded total RNA in combination with the ScriptSeq method for preparation of strand-specific RNA-Seq libraries. Furthermore, we observed some variation in rRNA contamination as found by Adiconis *et al.* [14]. However, by applying a strand-specific library preparation method we were able to differentiate between rRNA contamination due to insufficient depletion of rRNA and that due to contamination with rRNA probes. We found the latter to be the main source of rRNA contamination. Since the completion of the experimental part of this study, we have automated the Ribo-Zero and ScriptSeq technologies by implementing protocols for RNA from both FF and FFPE samples on a Sciclone NGS robot (Caliper/Perkin Elmer). Automation facilitates the use of the methods for studies of a large number of samples and has significantly reduced the fraction of rRNA reads in both FF and FFPE based RNA-Seq libraries. This suggests that insufficient mixing when adding the removal reaction to the washed and resuspended beads was the most likely cause of the observed contamination with rRNA removal probe sequences.

Paired statistical tests for significantly differentially expressed genes comparing benign and malignant colon tumours, revealed 471 and 1,207 genes to be affected in the FF and FFPE sets, respectively, of which 158 genes were common. In *the paired FF/FFPE bladder set* we observed a high correlation for a previously published [27] 12-gene expression signature for progression. We observed systematic effects on the RNA-Seq data caused by the fixation process and identified a common set of 1,494 genes whose expression profiles were significantly different between FF and FFPE samples among the three tissue sets of paired FF/FFPE specimens included. We found a reduced fraction of RNA-Seq reads mapping to exonic *vs* intronic regions in RNA-Seq data from FFPE compared with FF tissues. This has also been observed in previous studies [14], [15], [32] and has been attributed to degradation of the mature cytoplasmatic transcripts as a result of fixation. An alternative explanation for this observation could be that cross-linking of the mature transcripts to proteins results in their exclusion from the final purified RNA. The reduced exon representation in RNA-seq data derived from FFPE samples has an impact on the expression profiles, as shown in our study by the 1,494 genes whose expression profiles were significantly affected comparing FF and FFPE tissues across the three sample sets. We did find differences in the number and identity of the affected genes, when contrasting samples within the FF or FFPE sets, but did not find the genes included in the set of 1,494 FFPE-affected genes to be enriched. Although only a limited number of specimens were analysed, this provides important proof-of-principle evidence that similar RNA-Seq data expression profiles may be derived from tumour tissues, irrespective of whether FF or FFPE samples are studied. Before firm conclusions can be made concerning the possible application of RNA-Seq to FFPE-derived RNA, our preliminary data should be supplemented by larger studies based on more homogeneous sample sets including specimens with a wider range of storage times.

In general, we found a higher fraction of non-specifically mapped reads looking at NGS data from DNA compared with RNA analyses. Furthermore, there was a tendency for there to be a higher fraction of non-specifically mapped reads from FFPE compared with FF samples in DNA, but not RNA derived data. In general, the fraction of non-perfectly mapped reads was higher for RNA compared with DNA analyses; furthermore, this fraction was higher in analyses of FFPE compared with FF samples for both DNA and RNA. Increasing storage time tended to increase the fraction of both non-specifically and non-perfectly mapped reads in FFPE derived DNA, but had no apparent effect in FFPE derived RNA or in FF samples.

The data from the relatively few specimens we tested should naturally be viewed as preliminary. Moreover, in individual research projects, a decision must be made as to how much data deviation can be tolerated when comparing NGS profiling of FFPE tissues with the gold standard of FF analysis. With these provisos, our results are promising and suggest that NGS can be used to study DNA and RNA from FFPE specimens in both prospective and retrospective archive-based studies in which FF specimens are not available. This has important implications for opening the diagnostic pathology archives to high-throughput molecular profiling for both research and clinical purposes.

## Supporting Information

Figure S1 **The workflow in the project with specific focus areas highlighted.**
(TIF)

Figure S2 **The analytical workflow in CLC Genomics Workbench.**
(TIF)

Figure S3 **Insert sizes in DNA-Exome-Seq libraries from** ***the paired FF/FFPE colon set*** **.**
(TIF)

Figure S4 **Post-mapping results from the DNA-Exome-Seq libraries from** ***the paired FF/FFPE colon set*** **.**
(TIF)

Figure S5 **SNVs detected in DNA-Exome-Seq data from the paired FF/FFPE samples.**
(TIF)

Figure S6 **DNase vs DNase+ExoI treatment.**
(TIF)

Figure S7 **Pre mapping results from RNA-Seq of** ***the paired FF/FFPE bladder and prostate sets*** **.**
(TIF)

Figure S8 **Pre mapping results from RNA-Seq of** ***the storage time FFPE set*** **.**
(TIF)

Figure S9 **Post mapping results from RNA-Seq of** ***the paired FF/FFPE bladder and prostate sets*** **.**
(TIF)

Figure S10 **Post mapping results from RNA-Seq of** ***the storage time FFPE set*** **.**
(TIF)

Figure S11 **Fractions of duplicated reads in the RNA-Seq data sets.**
(TIF)

Figure S12 **FF vs. FFPE in RNA-Seq data from** ***the paired FF/FFPE colon, bladder and prostate sets*** **.**
(TIF)

Figure S13 **FF normal vs FF carcinoma in RNA-Seq data from** ***the paired FF/FFPE colon set*** **.**
(TIF)

Figure S14 **Benign vs malignant (FF or FFPE) in the RNA-Seq data from** ***the paired FF/FFPE colon set*** **.**
(TIF)

Table S1 **Detailed information on the sample sets.**
(XLSX)

Table S2 **Summarised information on isolated RNA and DNA from the different sample sets.**
(XLSX)

Table S3 **Summary of the DNA-Exome-Seq data from ten FF/FFPE pairs from** ***the paired FF/FFPE colon set*** **.**
(XLSX)

Table S4 **Gene ID and test statistics of the 1,494 genes found commonly affected by FFPE.** Gene ID and log2 fold-change (logFC.XXX), average log2-counts per million of all libraries in the set (logCPM.XXX), likelihood ratio statistics (LR.XXX), P values (PValue.XXX) and false discovery rate (FDR.XXX) for the colon (COL), prostate (PRO) and bladder (BLA) sets.
(XLSX)
