# Supplementary figures and images for: Next-Generation Sequencing of RNA and DNA Isolated from Paired Fresh-Frozen and Formalin-Fixed Paraffin-Embedded Samples of Human Cancer and Normal Tissue
Source: PLoS One. 2014 May 30;9(5):e98187. doi: 10.1371/journal.pone.0098187 (PMC4039489; doi:10.1371/journal.pone.0098187)

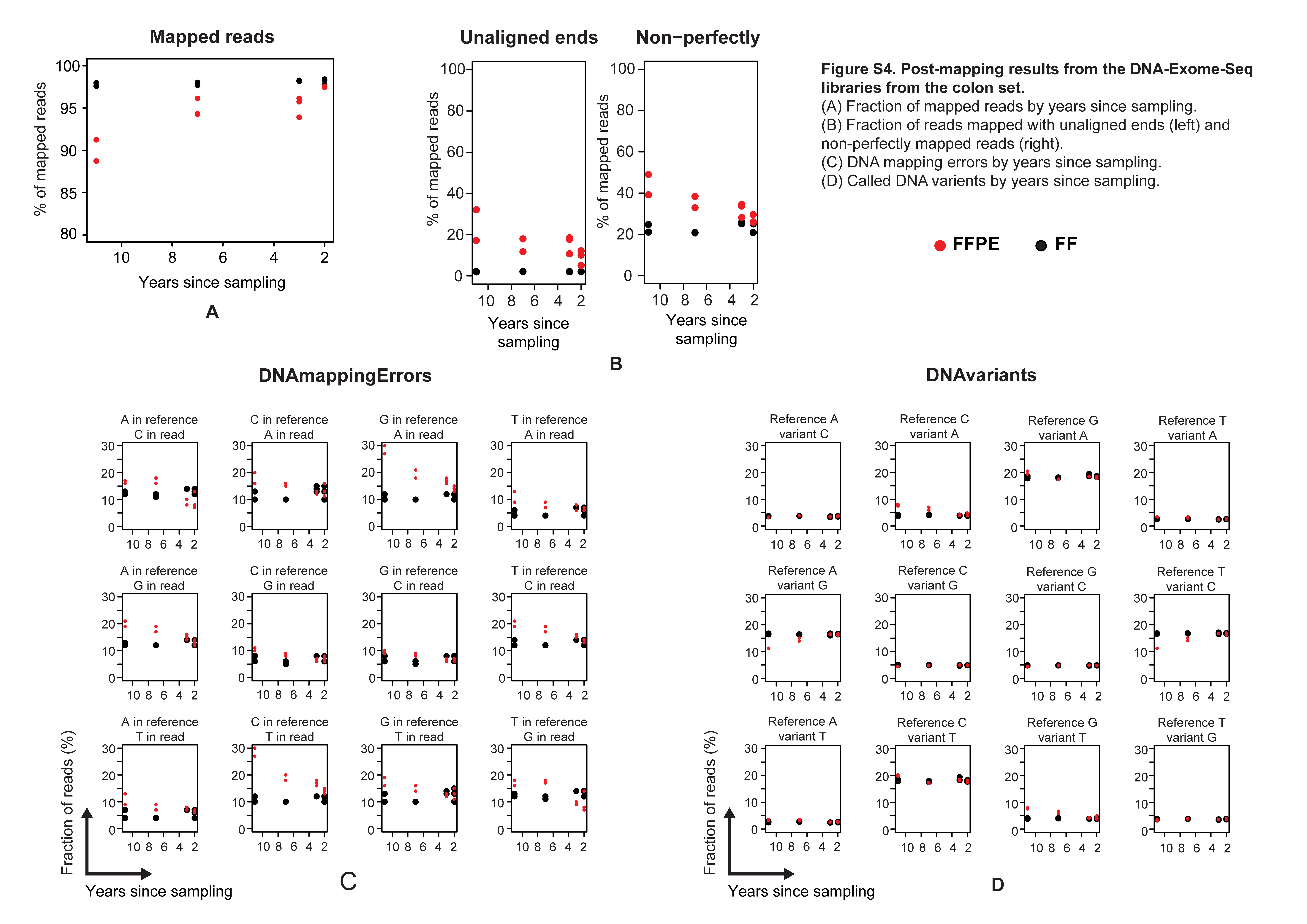

Supplement: Figure S4 — Post-mapping results from the DNA-Exome-Seq libraries from the paired FF/FFPE colon set . (TIF) [file pone.0098187.s004.tif]
